# Post-traumatic osteoarthritis: the worst associated injuries and differences in patients' profile when compared with primary osteoarthritis

**DOI:** 10.1186/s12891-023-06663-9

**Published:** 2023-07-12

**Authors:** Catrine Rangel Maia, Ricardo Fruschein Annichino, Marcelo de Azevedo e Souza Munhoz, Eduardo Gomes Machado, Evaldo Marchi, Martha Cecilia Castano-Betancourt

**Affiliations:** Faculty of Medicine of Jundiaí (FMJ), Rua Francisco Telles 250, Vila Arens, Jundiaí, SP 13202-550 Brazil

**Keywords:** Comorbidity, Hip, Injury, Knee, Post-traumatic osteoarthritis

## Abstract

**Background:**

The estimated prevalence of post-traumatic osteoarthritis (PTOA) is 10–12% and in this study 12.4%. Different knee and hip injuries have been identified as risk factors for PTOA, but there is no consensus regarding the most painful and disabling injuries. Identifying these injuries might help in the prevention of PTOA. Additionally, patients with PTOA have a higher risk for complications after arthroplasty than patients with primary OA, perhaps due to differences in the profile and comorbidity that might help to explain the difference. This work aims 1) to identify the most common past injuries associated with the most painful and disabling PTOA cases in non-athlete patients and 2) to compare the comorbidities and characteristics between PTOA and primary OA.

**Methods:**

Retrospective hospital-based cohort study with 1290 participants with joint complaints or who received arthroplasty. Medical records included demographic information, diagnosis, medication, smoking, alcohol history and comorbidities. Data from January 2012 orthopaedic consults till December 2019 was reviewed and had the type and date of injury, pain score by the numerical rating scale and walking disability. Odds Ratio (OR) and 95% confidence intervals are presented.

**Results:**

There were 641 cases with primary OA (65% females) and 104 with PTOA (61% males). Patients with PTOA were 7.5 years younger (*P* < 0.001), reported more alcohol consumption (*P* = 0.01) and had higher odds of osteoporotic fractures (OP) and psychosis than patients with primary OA (OR = 2.0, CI = 1.06–3.78 and OR = 2.90, CI = -0.91–9.18, respectively). Knee fractures were most common in males and hip fractures in females (31% and 37.5%, respectively, *P* < 0.005). The PTOA-associated injuries with the highest pain and disability scores were meniscal injuries and hip fractures. Besides, in the group with primary OA, there were more diabetes, hypertension and hypothyroidism cases than in PTOA. However, after adjustment, differences were only significant for diabetes (OR_ad_j = 1.78, CI = 1.0–3.2).

**Conclusions:**

Past meniscal injuries and hip fractures were the most relevant PTOA-associated injuries regarding pain and walking disability. This, together with differences in their profile when compared with primary OA, might help to decide the orthopaedic management of these injuries to prevent complications such as PTOA and recurrence, with appropriate preoperative planning, surgery choice and comorbidity treatment.

## Background

Osteoarthritis (OA) is a multifactorial disease that leads to articular cartilage degradation, affecting all joint components. OA is a slow, progressive and debilitating with a high prevalence in the active adult population, increasing worldwide [1]. OA is the third-largest contributor to the years lived with disability among musculoskeletal disorders, accounting for around 7.1% of this burden [2].

OA in younger populations is more commonly associated with traumatic injuries or sports practices. There has been an increase in post-traumatic osteoarthritis (PTOA) incidence in recent years due to the popularity of high-impact sports practices [3, 4]. Worldwide, 10–12% of all osteoarthritis cases are post-traumatic [5]. According to the Brazilian Society of Rheumatology (SBR), osteoarthritis is responsible for 7.5% of all absences from work in Brazil. It is the second disease among those that justify initial assistance (7.5%) or extension of sick leave (10.5%), being the fourth cause for retirement in the entire Brazilian population (6.2%). In those estimates, there is no information regarding the percentage corresponding to PTOA cases. There is also no information regarding Brazil's most relevant injuries or traumas that lead to PTOA. Most research in patients with PTOA has focused on a few types of knee injuries, principally anterior cruciate ligament and meniscal injury [6–8]. Limited epidemiologic data is available regarding other aspects and joints affected with PTOA, such as the hip.

Epidemiological differences between post-traumatic and primary OA are apparent. Subjects with traumatic knee OA have an earlier onset of the disease, various structural changes in the joints and biomechanical differences perceived during walking and running than primary OA cases [3, 4, 9–13].

Regarding complications and revision rates after total joint arthroplasty (TJA), PTOA presents an increased risk of complications. Infection, acute deep venous thrombosis, stiffness, and revisions occur more in patients with PTPA than in patients with primary OA without a known cause [14–17]. Contradictory, some authors found a lower prevalence of comorbidities in patients with PTOA than in those with primary OA and profiled patients with PTOA as “active patients with otherwise few comorbid health conditions”, which does not help understand the difference in complication and revision rates [18, 19].

Although considerable research investigates PTOA, few risk factors differentiate them from patients with primary OA. A risk profile for patients with PTOA will allow a more effective stratification of individual risk for PTOA, which is not possible yet [20]. With enough epidemiological knowledge, clinical trials may include patients at the highest risk of developing PTOA, facilitating markers and specific treatments for preventing PTOA. Few epidemiological studies compare comorbidities between PTOA and primary OA in the same population. Identification of these comorbidities might also help to decrease or prevent complications after arthroplasty.

Therefore, this work aims 1) to compare the comorbidities and characteristics of post-traumatic and primary OA cases and 2) to identify the most common injuries associated with the development of painful and disabling PTOA in non-athlete patients.

## Methods

### Study design and data source

This study is a retrospective hospital-based cohort study of men and women with joint complaints or who received a knee or hip arthroplasty. The hospital’s orthopaedic Department has a consulting unit dedicated to subjects with hip and knee pathologies requiring arthroplasty or revision. Medical records included demographic information, anamnesis, diagnosis, date, and joint surgery. In addition, laboratory measurements, medication, smoking and alcohol history, previous hospitalizations, past surgeries, consults to the hospital (previous or within the studied period), comorbidities and information from the orthopaedic consult, including the type of injury (if existed), date (if recalled), degree of pain and disability. Pain at each joint was registered using the numerical rating scale (NRS). Walking and stairs disability were evaluated as follows: without difficulty (0), with some difficulty (1), difficult or very difficult (2), and unable to do (3). The values were dichotomized (0/1) or added to be interpreted.

### Radiographic and other clinical variables

Most of the patients came to the orthopaedic consult referred from other services with a diagnosis of definite hip or knee OA, according to “The American College of Rheumatology” (ACR), or the “European League Against Rheumatism” (EULAR). At the orthopaedic department of the Hospital, all patients underwent hip or knee radiographs. Panoramic radiographs of both lower limbs, weight-bearing anteroposterior radiographs of the hip and knee and lateral knee joints were obtained. The observers were trained by an experienced researcher in OA (MCB) and advised by the hip and knee Orthopaedic specialists (EGM and MASM, respectively). Knee and hip radiographs were scored for the presence of osteophytes, joint space width and joint space narrowing by two independent observers (CRM and RFA) who used as a reference an atlas of individual radiographic features in OA [21].

A set of hundred seventy (170) radiographs of the joints: hips and knees, were graded based on Kellgren and Lawrence score (KL). Knee OA was defined as a KL score ≥ 2 (definite osteophytes and possible joint space narrowing) and hip OA as a definite joint space narrowing. Joint space narrowing was determined for the axial, lateral and superior sites of the hip or medial and lateral sites of each knee. The minimal joint space width was defined as the most narrowed site of the hip from the lateral, superior, and axial compartments. The space was measured with the help of the magnified feature of the program Pacs and Arya (version 21.3.1) from Pixeon. For the KL score, after the first set of 40 radiographs, the scores assigned by the two observers were evaluated using the intraclass correlation coefficient (ICC) for the joint space width and Cohen´s Kappa statistics for the osteophytes. The ICC for the minimal joint space was 0.79 (95%CI = 0.70–0.85) and the Kappa for osteophytes was 0.76.

Only verified musculoskeletal injuries that had led to continuous complaints or impairments were considered. The included injuries were severe enough to require a doctor’s consultation or hospital care and less severe self-reported traumas (e.g., ligament injuries). Injuries associated with signs of OA should be present at the same joint before OA symptoms develop. Injuries were classified as follows: Hip trauma (dislocation and fractures) and knee trauma (patellar dislocation, meniscal and ligament ruptures, knee fractures, and unknown mechanisms, principally due to sports practices). Comorbidities were identified, classified, or converted to the ICD-10 codes. Comorbidities listed on hospital discharge or by inter-consult request before the index surgery or orthopaedic consult. The following disease groups were analyzed: Cardiovascular disease, hypertension requiring medications, hypercholesterolemia, diabetes mellitus, mental comorbidity, osteoporosis, respiratory disease, deficiency anaemia and hypothyroidism. Analgesic use was also registered. We categorized alcohol consumption and smoking habit as former or current versus never used. Alcohol drinkers included those occasional or regular drinkers. Weight and height were measured, and Body Mass Index (BMI) was calculated.

This population is considered low-income, with 98% of the patients receiving a pension or a salary not higher than two minimum wages per family and registered in the public health Unique System (SUS). Occupations were housekeepers or homemakers, executive/desk jobs, textile, factory, cleaners, construction, and agriculture.

### Statistical analysis

The statistical plan included Analysis of variance (ANOVA) for continuous traits, logistic and linear regression, Odds Ratio (OR), and 95% Confidence Interval (CI) to analyze the association between primary vs post-traumatic OA risk factors. The Homogeneity of the variance was tested for continuous traits. If the assumption of homogeneity was violated, *P* values from non-parametric variables were obtained from Mann–Whitney U (2 samples). The median and interquartile range were presented instead of the mean and standard deviation (SD). *P* values for comparison from non-parametric variables with three or more groups were obtained from the Kruskal–Wallis test. For categorical variables, means were compared using Chi-square statistics were applied to determine statistically significant distributions between patients with PTOA or without PTOA. The statistically significant threshold was set at *P* ≤ 0.05 (2-tailed) and for categories with more than two groups (for example, mechanisms of injury), Bonferroni correction was applied. All risk factors or comorbidity analyses were adjusted for sex, age, and BMI. This study used IBM SPSS Statistics for Windows, version 25.0, for statistical analyses (IBM Corp, Armonk, NY, USA).

The Institutional Review Board of the Faculty (IRB) approved the study, and written informed consent was obtained from each participant.

## Results

### Prevalence of PTOA

**Fig. 1:**
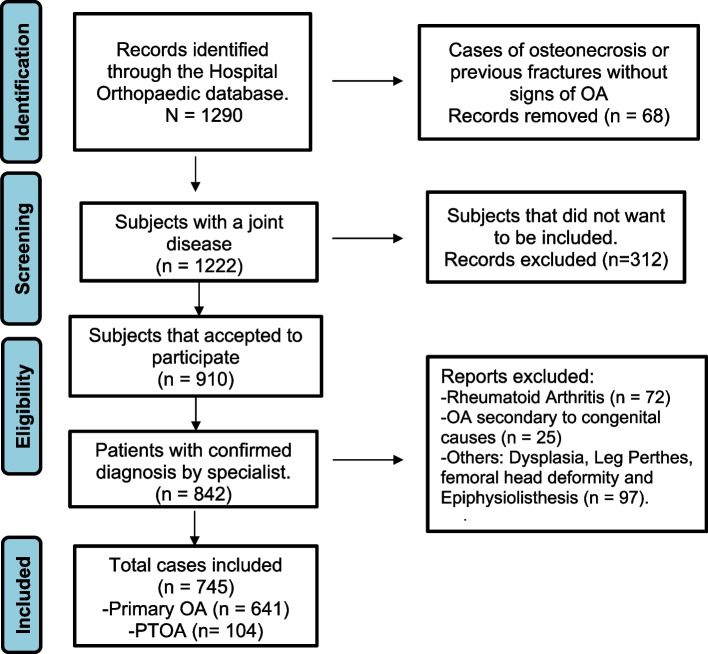
Flowchart for the included osteoarthritis cases

Thousand two hundred ninety (1290) patients attending the orthopedics service were diagnosed with a hip or knee joint disease and data on medical records. Sixty-eight patients were excluded for osteonecrosis or a previous fracture without OA evidence. In total, 842 patients had the following diagnosis: 641 patients (76%) with primary -OA, 104 patients with PTOA (12.4%), 72 with Rheumatoid arthritis (8.6%), and 25 patients (3%) with OA secondary to other joint abnormalities (hip dysplasia or dislocation, Leg Perthes, Femoroacetabular Impingement and epiphysiolistesis of proximal femoral). Among all patients with OA considering all causes mentioned in this paragraph, PTOA represents 13.6% of the cases (See Fig. 1).

### Type of injury, pain and disability

**Fig. 2:**
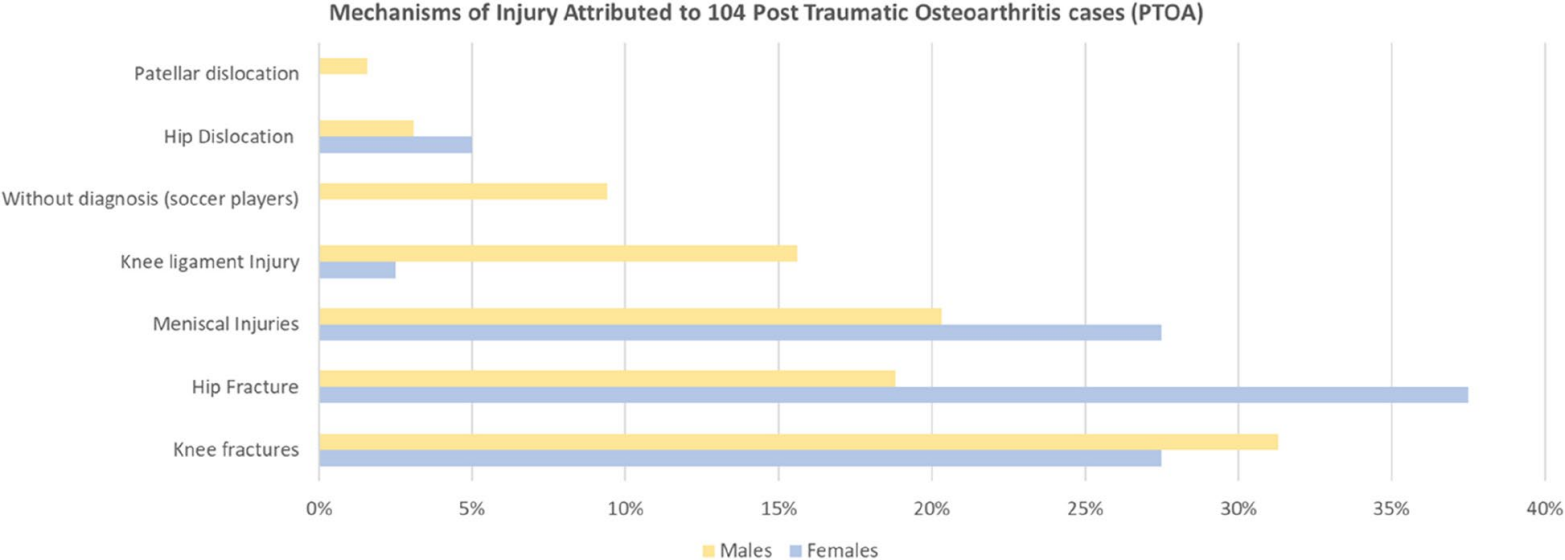
Associated injuries reported by post-traumatic osteoarthritis cases. Yellow bars for males and blue bars for females. In females, the most frequent injury was a hip fracture (37.5%), followed by meniscal tears and knee fractures with 27.5%. In males, it was knee fractures (31%), meniscal tears, and knee ligament injury, in that order. Significant differences in the most common injuries were between genders (*P* < 0.005)

Fractures were the most common injury for patients with PTOA; knee fractures in 30% of the knee-PTOA and femoral fractures (principally acetabular) in 26% of hip-PTOA cases (Fig. 2). Meniscal injuries and tears represented 23% of cases. Other types of injuries attributed to PTOA were ligament injuries (11%), hip dislocation (4%), and patellar dislocation (1%). Finally, in a group of former recreational soccer players (*n* = 6), there were no records of the injury and the subject did not recall the mechanism or diagnosis (Unknown mechanism, 6%). There were differences in the principal injuries associated with PTOA by gender; knee fractures were more common in males and hip fractures in females (Fig. 2, 31% and 37.5%, respectively).

**Fig. 3:**
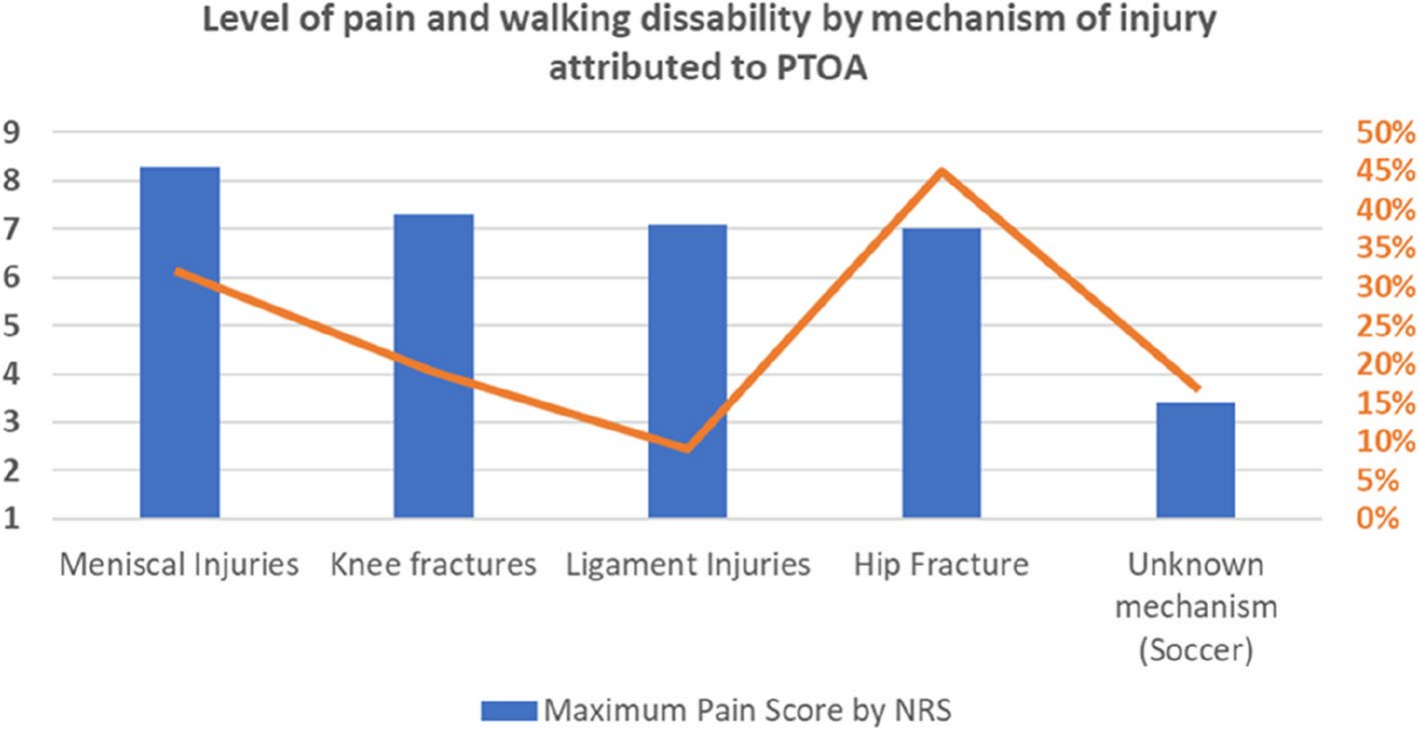
Pain and disability according to the associated PTOA-injury. The left axis (blue bars) shows patients’ maximum pain score using the Numerical Rating Scale (NRS, 0–10). The right axis (orange) shows the percentage of subjects with walking disabilities for each injury type. The orange line represents the percentage (%) of patients with difficulty walking or being unable. There were significant differences in pain scores between groups (P_Bonferroni_ = 0.024 for pain and P_Bonferroni_ = 0.14 for walking disability). Only categories with > 5 subjects were included in the analysis

Patients with PTOA and primary OA experienced a similar level of pain and disability (Median = 8 Q1-Q3 (6–10) for both, P (U Mann–Whitney) = 0.28 and mean for walking disability = 1.15 (OA) and 0.79 (PTOA), *P* = 0.45 for differences between the groups). Within the patients with PTOA, there were significant differences in the degree of pain according to the various injury mechanisms related to PTOA (Fig. 3, P_Bonferroni_ = 0.024). Patients with PTOA due to meniscal injuries had the highest pain score (Fig. 3, 8.3/10). In patients with PTOA due to past hip fractures, 45% had difficulty or were unable to walk, having two times higher odds for disability than patients with primary OA with only 32% (OR = 1.99, CI:0.93–4.26). Former recreational soccer players with an unknown injury mechanism had less pain and disability than other injury groups or primary OA cases (Fig. 3).

### Comparison between PTOA and Primary -OA cases

Most cases of primary osteoarthritis occurred in women (65%), and most cases of PTOA were in men (61%) (OR = 2.94, 95%CI: 1.92 – 4.50; *P* < 0.001). PTOA cases were younger than patients with primary osteoarthritis (Table 1, *P* < 0.001). Most patients with PTOA sustained the injury during adolescence or young adulthood. Therefore, most patients had no information on the database regarding surgeries, type of treatment or therapies received during the following week of the initial trauma. Until the last data collection date (December 2019), forty patients underwent TJA in the affected joint. Other thirty patients were scheduled for TJA, and the other group had difficulties performing the surgical procedure or decided not to perform the surgery. Patients with PTOA started with symptoms (pain) 6,5 years earlier than primary OA patients (47.8 (± 1.2) and 54.3 (± 0.48), respectively, *P* < 0.001). The knee was the most affected joint in PTOA and OA cases (73% and 70%, respectively). Alcohol consumption was more frequent in PTOA than in primary OA cases (OR_adj_ = 1.83, 1.4–2.94, *P* = 0.01). However, after adjustment with gender, age, and BMI in the model, it was not significant (Table 1, Padj = 0.89).

**Table 1:** Descriptive statistics of the studied population

**Characteristics**	**Univariate analyses**					
	**OA (*n* = 641)**	**PTOA (*n* = 104)**	***P*-value**	**OR**	**95% CI**	***P*-value**^ +^
Age: Mean (SD)	67.2 (0.44)	59.7 (1.02)	** < *0.001***^***u***^			
AGE CATEGORIES < 60 years	138 (215)	51 (49%)	** < *0.001***	6.28	3.46 – 11.4	** < *0.001***
Sex Male	226 (35)	64 (61)	** < *0.001***	2.94	1.92 – 4.50	** < *0.001***
BMI (kg/m^2^)	30.3 (0.23)	29.3 (0.57)	0.096			
Obesity (BMI > 30)	30.2 (47.1)	42 (40.4)	0.202	0.80	0.51 – 1.25	0.32
**Adjusted analyses**						
Occupation: Desk jobs	109 (17)	35 (34)	** < *0.001***	2.01	1.24 – 3.27	***0.005***
Knee joint	459 (72)	73 (70)	0.60	0.60	0.38 – 0.93	***0.02***
Hip Joint	182 (28)	31 (30)				
Hypertension	442 (69)	58 (57)	***0.013***	0.95	0.58 – 1.54	0.83
Hypercholesterolemia	233 (37)	29 (30)	0.15	0.86	0.53 – 1.40	0.54
Diabetes Mellitus	183 (29)	17 (16)	***0.009***	0.56	0.31 – 0.99	***0.05***
Osteoporosis*	179 (28)	69 (66)	** < *0.001***	2.00	1.06 – 3.78	***0.03***
Smoking^**^	183 (29)	37 (36)	0.15	0.91	0.57 – 1.76	0.69
Alcohol use^**^	111 (17)	29 (28)	***0.01***	0.96	0.56 – 1.65	0.89
Mental Comorbidities	75 (12)	13 (13)	0.82	1,28	0.65 – 2.51	0.47
Psychosis	11 (2)	5 (5)	***0.03***	2.90	0.91 – 9.18	0.07
Analgesics use	474 (74)	66 (64)	***0.026***	0.63	0.40 – 1.00	***0.05***
Walking Disability (Bin)	500 (78)	71 (68)	***0.03***	0.76	0.47 – 1.22	0.25
Stairs difficulty (Bin)	562 (88)	84 (81)	***0.05***	1.06	1.02 – 1.11	0.28
Hypothyroidism	83 (13)	6 (6)	***0.036***	0.66	0.27–1.66	0.37
Anemia	19 (3)	2 (2)	0.57	0.66	0.18–2.8	0.66
Pulmonary dis	51 (8)	5 (5)	0.33	0.62	0.24–1.60	0.33
Cardiovascular dis	79 (12)	10 (10)	0.43	1.00	0.47 – 2.17	0.99

Univariate analysis: the values ​​are means or numbers and standard error (SD) or percentages for categorical variables in parentheses. (BMI) = Body mass index (weight in kilograms / (height in meters^2^). P^+^= P value from analyses adjusted for age, sex and BMI. The values ​​presented are Odd Ratios (OR) with a 95% confidence interval (CI). Reference = primary OA cases

^***u***^ Age was compared between the groups using Mann–Whitney U (2 samples)

^*^Exclusion of fractures associated with PTOA

^**^Past and current versus never used. Bold and italics for significant *P* values

^***^Psychosis includes bipolar disorder and schizophrenia cases

Diabetes was less frequent in PTOA than in primary OA cases even after adjustment for.age, sex, and BMI (Table 1, OR = 0.56. 95%CI: 0.31–0.99). There were more subjects with hypertension and hypothyroidism in the group with primary OA than PTOA (Table 1, unadjusted *P* < 0.05). Differences were not significant after adjustment (Table 1, *P* = 0.83 for hypothyroidism and *P* = 0.37 for hypertension). Desk jobs were the past more frequent occupations for patients with PTOA than primary OA (34% vs 17%, Table 1, OR = 2.01, 95%CI = 1.24–3.27). Patients with PTOA had higher odds of OP fractures than patients with primary OA, even after excluding the initial PTOA-related fracture (Table 1, OR = 2.0, CI = 1.06–3.78). Patients with PTOA had 2.9 higher odds of having psychosis (from bipolar disorder or schizophrenia), borderline significant (Table 1, OR = 2.90, CI = -0.91–9.18). Painkillers were less used in the group with PTOA than primary-OA (OR = 0.56, 95%CI: 0.40–1.0, Table 1, *P* = 0.05). All other factors were similar between PTOA and primary OA after adjusting for age, sex, and BMI (Table 1).

## Discussion

This study found a prevalence of PTOA of 12.4%, higher for the knee than the hip, with 9% and 3.4%, respectively. These rates are like those reported in international literature, with 10–12% of OA cases considered PTOA [5, 22–24]. Persons with PTOA account for nearly 12% of all cases of symptomatic OA in the USA [5]. Posttraumatic OA of the hip represented approximately 2% of all cases of hip OA and 20% in military personnel [25]. There are no more updated reports of the prevalence of PTOA.

Although PTOA is less common than primary OA, it becomes of particular interest, considering it affects a younger population and increases the risk of complications after arthroplasty. Younger populations sustain more injuries than older, and a knee injury increases 4.2 times more the chances of developing OA compared to those without a history of knee injury [23]. Most patients suffered index injury or initial trauma in young adulthood before 40 years, with osteoarthritic pain symptoms beginning seven years earlier than primary OA cases that started with joint pain around 54 years of age. On average, in the forties, the first sign of knee osteoarthritis appears in patients with combined knee injuries and 50 years for those with only a meniscal injury [26, 27]. Consequently, some of these patients require arthroplasty much earlier than OA cases and might present complications increasing the possibility of disability and early retirement.

In this study, men were more affected by traumas than females, principally knee fractures and meniscal tears. Meniscal tears are roughly twice as common in men than women and more likely to be traumatic in males than in females [28–31]. Patients with meniscal injuries reported the highest pain score of all PTOA cases. Regarding the hip joint, past hip fractures were the most common for hip PTOA. These patients had a high pain score, similar to ligament injury or knee fracture and had more difficulty walking or climbing stairs than patients with other trauma.

One of the risk factors for PTOA in this study was a history of fractures. Patients with PTOA sustained more fractures in the past than those with primary OA, even after excluding the causal trauma. This factor might be due to undiagnosed osteoporosis, principally in females with hip fractures, or riskier behaviour in men associated with an increased chance of sustaining an injury [32]. There was a higher level of smoking, alcohol intake and psychosis in patients with PTOA compared to patients with primary OA Alcohol consumption and psychosis increase various forms of risk-taking behaviour and is associated with a significant increase in osteoporotic and hip fracture risk [33, 34].

This study is one of the few that compare comorbidities between patients with PTOA and primary OA. The higher prevalence of smoking, alcohol use and psychosis in the PTOA group might help explain the worse outcomes after arthroplasty in patients with PTOA. Patients with PTOA are at increased risk for infection, stiffness, and revision than patients with primary OA [14, 15, 35]. Alcoholism is associated with higher wound infection rates and delay in wound closure [36]. Smoking also increases the risk of complications after surgery, including impaired wound and bone healing [37]. Having a psychiatric disorder is considered a risk factor for complications after Total Knee Arthroplasty, including extensor mechanism rupture, periprosthetic infection, fractures, and increased revision rate [38].

In contrast, patients with primary OA have a higher prevalence of hypertension, hypothyroidism, diabetes, and obesity. This aspect emphasizes the metabolic component of primary OA and its higher number of comorbidities than PTOA. However, we should be cautious in interpreting these associations because they do not necessarily imply causation. There is a need for further research to establish causal relationships and investigate potential underlying mechanisms.

Several genetic variants have been mentioned as risk factors for OA, and it seems that they exert a similar genetic effect on the risk of hip or knee OA in PTOA and non-traumatic cases of clinically severe OA leading to TJA [39]. Our study did not include a comparison of genetic variants between PTOA and primary OA cases but, this topic remains interesting for future research.

This study has several strengths and limitations. First, illustrating the differences between PTOA and OA has important clinical implications. OA is associated with multimorbidity and polypharmacy [40]; however, differences exist between comorbidities in PTOA and primary OA. Comorbidity prevention programs in OA should first consider which type of OA the patient has and test for the most prevalent comorbidities for each group. There is a need for more prevention of comorbidities and personalized care in patients with OA. Weight control and injury prevention are the most effective primary OA and PTOA prevention strategies, respectively. Identification of the injuries that produce more pain and disability is crucial, considering that no treatments are currently available to prevent further joint degeneration.

The results of this study contribute to outlining a clear profile of patients with PTOA, including differences in comorbidities with primary OA, that might help to take preventive actions to decrease the number of complications after arthroplasty in this group. It is known that patients with PTOA have more complications related to infections after arthroplasty, it would be possible to reduce this type of complication with prevention efforts regarding osteoporosis, smoking, alcohol intake, and psychiatric referrals in this population.

History of fractures, male gender, psychotic disease, regular alcohol intake and smoking are possible risk factors for trauma and subsequent PTOA.

According to data from the Ministry of Health (DATASUS), osteoarthritis affects 30 million people in Brazil, accounting for, on average, 30% to 40% of rheumatology consultations across the country (ww.saude.gov.br). Guaranteed in the Federal Constitution, SUS is the only public health system in the world that serves more than 190 million people, 80% of whom depend exclusively on public services for any health care (unasus.gov.br). According to statistics from the Ministry of Social Security, in Brazil, OA is currently the third leading cause of leave from work and the third leading cause of premature retirement due to disability (www.gov.br). Most of our cohort's patients consulted with orthopaedic surgeons for severe OA, requesting surgical treatment. Therefore, the subject included in this study might represent only patients with the last stage of OA, limiting the generalisation of our findings.

The selection criteria was limited to patients with a confirmed diagnosis of OA to reduce misclassification bias. In addition, some patients self-reported the trauma and type of injury with the risk of recall bias. Additionally, the causal trauma was not verified for some patients and when it occurred was not investigated by imaging. Ascertainment biases may be present due to misdiagnosis of the injury type or mechanism and comorbidity miscoding. In general, the number of PTOA cases is reduced compared to primary OA; principally, the number of patients with hip-PTOA was limited compared to knee-PTOA, which does not allow stratification or further comparisons. Finally, we have all the limitations given by the retrospective design of this study and the study is limited to the findings of this cohort. A multicenter or more powered study would be beneficial to confirm these findings.

## Conclusions

In conclusion, PTOA represents around 12.4% of all OA cases. In this population, injuries in patients with knee or hip PTOA were similar to those reported in other populations, such as meniscal tears, ligament injuries, and knee and hip fractures. Subjects with PTOA have these characteristics: younger age, more males with more cases of psychosis, smoking and a higher alcohol intake than subjects with primary OA. It is essential to highlight that these associations do not imply causation. Patients with PTOA and past meniscal injuries reported the highest pain score, and those with hip fractures reported the highest disability level. Patients with primary OA had a higher prevalence of hypertension, diabetes and hypothyroidism than subjects with PTOA.

Interventions to reduce complications in patients with OA should consider the profile of the patients given by the type of OA Patients with PTOA might benefit from a program to reduce alcohol consumption and smoking, at least during the preoperative and postoperative periods. They might also benefit from psychological counselling or psychiatric consultation to work on these risk factors and verify if those patients diagnosed with a mental psychotic disorder are receiving adequate treatment. Controlling these risk factors might prevent complications after arthroplasty (if needed) and decrease the risk of new traumas or injuries.

## Data Availability

The datasets used and/or analyzed during the current study are available from the corresponding author on reasonable request.

## Abbreviations

Adj: Adjusted
Bin: Binary
BMI: Body Mass Index
CI: Confidence Interval
OA: Osteoarthritis
PTOA: Post-traumatic osteoarthritis
NRS: Numerical Rating Scale
OR: Odds Ratio
TJA: Total Joint Arthroplasty
TKA: Total Knee Arthroplasty
